# G3 2022 Reviewer Index

**DOI:** 10.1093/g3journal/jkac298

**Published:** 2022-11-15

Below is a list of individuals who have reviewed manuscripts for G3: Genes | Genomes | Genetics during the preparation of Volume 12 (2022). The aim of G3: Genes | Genomes | Genetics is to communicate high-quality, well-described, useful research, across all areas of genetics and genomics. To succeed, we must recognize what meets our criteria for publication, making sure the presentation is accurate, unambiguous, and easily readable. For this, we depend heavily upon our reviewers, who perform this indispensable service without reward. The quality of the journal strongly reflects the quality of their efforts. We apologize to any reviewer whose name has been inadvertently omitted, and we are sincerely grateful to all.

Abalde, Samuel

Abdelraheem, Abdelraheem

Achaz, Guillaume

Adams, Keith

Aguate, Fernando

Ahmed, Yashi

Ahmed, Shawn

Albert, Frank

Allen, Scott

Allier, Antoine

Alspaugh, J.

Alvarez-Ponce, David

Andersen, Erik

Anderson, Kirk E.

Anderson, Matthew

Anderson, Amy

Andersson, Leif

Aoun, Meriem

Appel, Bruce

Araújo, Sofia

Arbeitman, Michelle

Arboleda, Valerie

Arimoto, Asuka

Armstrong, Ellie

Atallah, Joel

Atanda, Sikiru

Aulitto, Martina

Avalos, Arian

Avni, Raz

Babitzke, Paul

Bachvaroff, Tsvetan

Baer, Charles

Baier, Margarete

Bancic, Jon

Bandillo, Nonoy

Barbash, Daniel

Barber, Amelia

Barkman, Todd

Barrios, Arantza

Barutcu, Rasim

Basrai, Munira

Battey, C. J.

Battlay, Paul

Baud, Amelie

Baxter, Ivan

Becker, Doreen

Beever, Jon

Beier, David

Beitel, Greg

Bélanger, Sébastien

Beliveau, Brian

Bell, Avery

Bellone, Rebecca

Benning, Christoph

Bertho, Sylvain

Bertolini, Edoardo

Besson, Mathieu

Betran, Esther

Bianchi, Laura

Bier, Ethan

Biscarini, Filippo

Blackmon, Heath

Bland, Michelle

Bobay, Louis-Marie

Böhne, Astrid

Bonetti, Diego

Booker, Tom

Bordenstein, Seth

Boria, Robert

Bosco, Giovanni

Bouwman, Aniek

Bracewell, Ryan

Braendle, Christian

Brandl, Christopher

Brandvain, Yaniv

Brault, Charlotte

Bresolin, Tiago

Brookfield, John

Brown, Susan

Brush, George

Bryan, Glenn

Bryant, Astra

Buell, C. Robin

Buffalo, Vince

Buratowski, Stephen

Burga, Alejandro

Burgess, Shawn

Bushman, Bradley

Cai, Rui

Caissard, Jean-Claude

Cannon, Steven

Cantor, Rita

Castellano, David

Castillo Vardaro, Jessica

Caudron, Maiwen

Cazares, Adrian

Chakraborty, Mahul

Chan, Ting-Fung

Charette, Michael

Chari, Tara

Chee, Peng

Chekan, Jonathan

Chen, Naichong

Chen, Julian

Cheng, Liang

Chin, Jason

Chow, Clement

Chua, Gordon

Chuong, Cheng-Ming

Clement, Tracy

Clowney, E. Josephine

Coffing, Gabrielle

Cohen, Zach

Cole, Charles

Conover, Justin

Cook, David

Cook, Kevin

Cooper, Terrance

Cordellier, Mathilde

Cormack, Brendan

Costa-Neto, Germano

Costilla, Roy

Coughlan, Jennifer

Coutinho, Luiz Lehmann

Couto Alves, Filipe

Cox, Rachel

Cram, Erin

Croll, Daniel

Crook, Nathan

Crouch, Jo

Crown, Nicole

Csankovszki, Györgyi

Cuevas, Jaime

Cuomo, Danila

Cuomo, Christina

Cussiol, José Renato

Dadousis, Christos

Dapper, Amy

Davidson, Alan

Davis, Brian

Dawe, R.

De, Sandip

De la Cova, Claire

De Oliveira, Andre Luiz

De Vries, Charlotte

Dearden, Peter

Debets, Fons

DeFalco, Tony

Derežanin, Lorena

Des Marais, David

Deshpande, Girish

DiAngelo, Justin

Diaz, Raul

DiCara, Francesca

DiCenzo, George

Dickhout, Jeffrey

Dickinson, Mary

Ding, Haidong

Dolezal, Tomas

Domonkos, Svab

Dong, Zhiguo

Donze, David

Draper, Bruce

Dreger, Dayna

Dumont, Beth

Dunn, Ian

Durvasula, Arun

Dyer, Paul

Earl, Julie

Ebel, Emily

Edwards, Jode

Ehringer, Marissa

Ekblom, Robert

Ekenstedt, Kari

Enard, David

Endelman, Jeffrey

Engelstaedter, Jan

Estelle, Mark

Evans, Jay

Fanara, Juan

Farabaugh, Philip

Farrer, Rhys

Fay, Justin

Ferchaud, Anne-Laure

Fernandes, Samuel

Fierst, Janna

Fischer, Sylvia

Fischer, Andreas

Flames, Nuria

Fok, Ellis

Forche, Anja

Formenti, Giulio

Forsythe, Evan

Freitag, Michael

Frøkjær-Jensen, Christian

Fukuyama, Masamitsu

Gáliková, Martina

Gallone, Brigida

Gao, Guangtu

Garcia-Guerra, Azalea

Garfield, Victoria

Garona, Ana

Gartner, Anton

Gatins, Remy

Gaudet, Muriel

Gauthier, Jérémy

Georgiev, Pavel

Gérard, Pierre

Giaimo, Stefano

Gignoux, Christopher

Gill, Kulvinder

Girirajan, Santhosh

Glanzmann, Brigitte

Gloss, Andrew

Gluck-Thaler, Emile

Goldman, Gustavo

Goloborodko, Anton

Govind, Shubha

Graham, JInko

Grant, Barth

Graze, Rita

Greenwald, Iva

Gresham, David

Grewal, Savraj

Griffin, Erik

Gromak, Natalia

Grover, Corrinne

Guerreiro, Marco

Guet, Calin

Guiguen, Yann

Gupta, Bhagwati

Gutierrez, Alejandro

Gutiérrez, Juan Pablo

Haag, Eric

Hall, Sarah

Hallem, Elissa

Hammond, Chrissy

Hane, James

Hanotte, Olivier

Hansen, Allison

Hart, Craig

Hashiguchi, Yasuyuki

He, Feng

Heiman, Maxwell

Henderson, Ian

Henke, Katrin

Henson, Michael

Heras, Joseph

Hieter, Philip

Holland, James

Holliday, Jason

Holmes, Scott

Hom, ERIK

Hori, Tiago

Hoskins, Aaron

Hou, Jing

Howard, Reka

Hsueh, Yen-Ping

Hu, Hao

Hu, Patrick

Hu, Guanjing

Huang, Sanwen

Huang, Cheng-Yang

Huberman, Lori

Hughes, Lee

Huynh, Bao Lam

Ikui, Amy

Irish, Vivian

Ishikawa, Akira

Janss, Luc

Janzen, Garrett

Jaramillo-Lambert, Aimee

Jarquin, Diego

Jasper, Heinrich

Javal, Marion

Jenkins, Paul

Jensen, Sarah

Jiang, Jiming

Jighly, Abdulqader

Johansen, Kristen

Johansson, Anna

Johnsson, Martin

Johnston, J. Spencer

Johri, Parul

Jones, Corbin

Jongens, Tom

Kadyrov, Farid

Kardos, Marty

Karp, Xantha

Karpe, Snehal

Kause, Antti

Kaya, Yalcin

Ke, Wenfan

Kellogg, Elizabeth

Kim, Bernard

Kim, John

Kirienko, Natalia

Klebba, Phillip

Klump, Kelly

Koch, Evan

Koop, Ben

Kouvelis, Vassili

Kozik, Alexander

Krabbenhoft, Trevor

Krasovec, Marc

Kronstad, James

Krugman, Tamar

Kupczok, Anne

Kupiec, Martin

Kwon, Jae Young

L'Etoile, Noelle

L'Hernault, Steven

Labroo, Marlee

Lai, Eric

Lai, Zhi-Chun

Lambert, Iain

Lamberti, Pedro

Landis, Jacob

Langley, Charles

LaRocque, Jeannine

Lawrence, Erica

Lazetic, Vladimir

Lee, Hangnoh

Lee, Yuh Chwen

Lee, Cheng-Ruei

Lee, Siu Sylvia

Leeb, Tosso

Legarra, Andres

Legras, Jean-Luc

Lei, Li

Leppälä, Kalle

Lew, Daniel

Lewis, Zachary

Li, Haipeng

Liachko, Nicole

Lian, Ling

Liang, Chengzhi

Liao, Yi

Lima, Dayane

Lin, Feng

Lindgren, Gabriella

Lindsey, Amelia

Link, Vivian

Link, Christopher

Liu, Bobin

Liu, Zhanjiang

Liu, Yi

Lloyd, Andrew

Lobner-Olesen, Anders

Lofgren, Lotus

Lopes, Marcos

Lopes-Marques, Mónica

Lopez-Cruz, Marco

Loraine, Ann

Lorenz, Aaron

Lou, Hua

Louha, Swarnali

Luan, Qifu

Lucas, Stuart

Lusetti, Shelley

Lynch, Rachel

Mabry, Makenzie

Maccaro, Jessica

MacColl, Andrew

Macias, Vanessa

Mackay, Ian

MacQueen, Alice

Macqueen, Daniel

Maduro, Morris

Mahony, Shaun

Mains, Paul

Makunin, Alex

Maloof, Julin

Mandel, Jennifer

Manhart, Carol

Marigorta, Urko

Marlow, Florence

Marrec, Loïc

Martínez-Gómez, Pedro

Martini, Johannes

Maside, Xulio

Masson, Patrick

Mata, Juan

Mathers, Thomas

Mathews, Sarah

Matthews, Ben

Mattiazzi Usaj, Mojca

Matus, David

Maxwell, Karen

May, Georgiana

Mazourek, Michael

McCallion, Andrew

McConkey, Brendan

McElroy, Kyle

McEwan, John

McGuigan, Katrina

McNeill, Elizabeth

Mendoza, Manuel

Meneghini, Marc

Menon, Mitra

Mera, Paola

Merot, Claire

Merritt, Thomas

Meyer, Diogo

Militello, Kevin

Miller, Eric

Miller, Danny

Misztal, Ignacy

Mitchell, Aaron

Moerman, Donald

Mongue, Andrew

Montgomery, Tai

Montoliu-Nerin, Merce

Morano, Kevin

Moriyama, Etsuko

Morota, Gota

Morrell, Peter

Morse, Randall

Morton, Brian

Mosca, Timothy

Mott, Richard

Moye-Rowley, W.

Mukherjee, Tina

Munoz, Patricio

Murphy, William

Natvig, Donald

Neiman, Maurine

Nelson, Thomas

Nettelblad, Carl

Neyhart, Jeffrey

Noble, Suzanne

Norris, Adam

Nowrousian, Minou

O'Donnell, Michael

O'Meara, Teresa

O'Neill, John

O'Rourke, Eyleen

Ogunremi, Dele

Ojeda, Dario

Olatoye, Marcus

Osipova, Ekaterina

Otyama, Paul

Padilla García, Nelida

Pak, Stephen

Palaiokostas, Christos

Palero, Ferran

Palmer, Daniela

Palti, Yniv

Palu, Rebecca

Pan, Weijun

Pantha, Pramod

Papp, Balazs

Park, Joong-Ki

Parker, Clarissa

Parkin, Isobel

Parsch, John

Parsons, Jeremy

Pasaniuc, Bogdan

Pathak, Narendra

Patton, Elizabeth

Pearce, Stephen

Pelaez, Julianne

Pélissié, Benjamin

Peris, David

Petrella, Lisa

Piantadosi, Anne

Pickett, Brandon

Pinto, Brendan

Pividori, Milton

Plomion, Christophe

Podrabsky, Jason

Pombo, Marina

Pook, Torsten

Pool, John

Porceddu, Andrea

Potter, Christopher

Prendergast, James

Pritchard, Victoria

Puddu, Fabio

Purugganan, Michael

Qiao, Shiyan

Radanović, Aleksandra

Ragsdale, Aaron

Rai, Madhulika

Ramesh, Balan

Rando, Halie

Rangan, Prashanth

Rankin, Catharine

Rasheed, Awais

Read, Andrew

Rebay, Ilaria

Rebelo, Sandra

Redhai, Siamak

Regan, Tim

Reid, Noah

Reiner, David

Reinke, Valerie

Reiter, Larry

Rhodes, Johanna

Rice, Brian

Rieder, Leila

Riley, Bruce

Rincent, Renaud

Rissland, Olivia

Roalson, Eric

Robinson, Gene

Rocheleau, Christian

Rogel-Gaillard, Claire

Rogers, Rebekah

Rollins, Jeffrey

Rondeau, Eric

Ronnberg-Wastljung, Ann-Christin

Rouse, Matthew

Rousselle, Marjolaine

Rowe, Annette

Roy, Sourav

Roy, Richard

Ruan, Yuefeng

Runcie, Daniel

Rusan, Nasser

Rusche, Laura

Rutkoski, Jessica

Ruttink, Tom

Ruvinsky, Ilya

Sackton, Timothy

Saha, Surya

Sahana, Goutam

Salzberg, Steven

Sampaio, Jose

Samuk, K.

Sánchez Pérez, Raquel

Sandve, Simen

Santos, Renato

Saura, Maria

Schield, Drew

Schrider, Daniel

Schroeder, Jeremy

Schumacher, Julia

Scotti, Ivan

Sederoff, Ronald

Seetharam, Arun

Seidel, Hannah

Serrano-Saiz, Esther

Servin, Bertrand

Seymour, Danelle

Shahrestani, Parvin

Shakes, Diane

Shalizi, Mohammad

Shannon, Laura

Sharakhova, Maria

Sharma, Ram Kumar

Shavrukov, Yuri

Shewaramani, Sonal

Shore, David

Shukla, Harsh

Shull, James

Shultz, Allison

Signor, Sarah

Simon, Rebecca

Simons, Yuval

Singh, Sadhana

Singh, Ravinder

Sivasankaran, Sathesh

Skiadas, Petros

Sloan, Daniel

Slot, Jason

Smith, Clare

Smith, Seth

Smith, Harold

Smith, Shavannor

Snodgrass, Samantha

Sockanathan, Shanthini

Sokol, Nicholas

Solares, Edwin

Solari, Katherine

Soltis, Pamela

Somorjai, Ildiko

Sood, Salej

Soto, Teresa

Sousa, Ana

Southall, Tony

Spana, Eric

Sparks, Michael

Spear, Melissa

Sprecher, Simon

Spribille, Toby

Stajich, Jason

Stevens, Lewis

Stevison, Laurie

Stram, Daniel

Stuckert, Adam

Studholme, David

Su, Baofeng

Sudmant, Peter

Sun, Wei

Sung, Way

Supek, Fran

Surtees, Jennifer

Syed, Zulfeqhar

Tamplin, Owen

Tao, Qiqing

Taubert, Stefan

Tennessen, Jason

Teotónio, Henrique

Tessele, Augusto

Teterina, Anastasia

Thompson, Andrew

Timp, Winston

TING, CHAU-TI

Tittes, Silas

Tixier-Boichard, Michele

Townsend, Jeffrey

Tran, Elizabeth

Tschopp, Patrick

Tu, Zhijian

Ueltzhöffer, Kai

Updike, Dustin

Usher, Jane

Van Belleghem, Steven

Van Dijck, Patrick

Van Doren, Mark

Van Lieshout, Natascha

Vannini, Alessandro

Vaughn, Justin

Verta, Jukka-Pekka

Vicoso, Beatriz

Vieira, Jorge

Villanea, Fernando

Volk, Talila

Voronina, Ekaterina

Wachi, Nakatada

Wade, Paul

Walters, James

Wang, Juntao

Wang, Cuiling

Wang, Baohua

Wang, Diane

Wang, Lizhi

Wang, Ping

Ward, Robert

Wares, John

Warrior, Rahul

Waterhouse, Robert

Watts, Jennifer

Webster, Tim

Webster, Amy

Wei, Kevin

Wei, Shu-Jun

Whibley, Annabel

Whitehead, Mark

Whiteway, Malcolm

Whitworth, Cale

Wickner, Reed

Williams, Ernest

Wisecaver, Jennifer

Wolfe, Joanna

Wolfe, Thomas

Wong, Darren

Woodruff, Gavin

Worthen, Jennifer

Worthington, Margaret

Wright, Stephen

Wu, Zhiqiang

Xiaofei, Yang

Xiong, Ai

Xu, Jianping

Xu, Yongjie

Yalcin, Binnaz

Yamamoto, Shinya

Yamasaki, Yo

Yan, Huihuang

Yang, Feng

Yanowitz, Judith

Yao, Tao

Ye, Wuwei

Yu, Jun

Yu, Fuqiang

Yuan, Daojun

Yusuf, Leeban

Zaman, Luis

Zamanian, Mostafa

Zaugg, Judith

Zetka, Monique

Zhang, Gaotian

Zhang, Liusuo

Zhao, Shuai

Zhao, Chuanji

Zhao, Chunjiang

Zhou, Baoliang

Zhou, Xiao-Jun

Zhou, Yongfeng

Zhu, ChaoDong

Zhuang, Zhanwei

Zimin, Aleksey

Zirin, Jonathan

Zou, Fei

